# A Measure of Depression in a Modern Asian Community: Singapore

**DOI:** 10.1155/2012/691945

**Published:** 2012-10-09

**Authors:** Weining C. Chang, Jessie Bee Kim Koh

**Affiliations:** ^1^Institute of Mental Health, Singapore 539747; ^2^Department of Clinical Sciences, Graduate School of Medicine, Duke-National University of Singapore, Singapore 169857; ^3^Cornell University, Ithaca, NY 14850, USA

## Abstract

The construct validity of two depression measures, Zung's Self-rating Depression Scale (SDS) and the Asian Adolescents Depression Scale (AADS), was investigated. Three studies were conducted using two samples collected in two stages, and the results were used to construct the Asian Depression Scale (ADS). Participants responded to the SDS and AADS in random order of presentation during stage 1; two months later, validation variables were collected. Study 1 found that the SDS is a reliable and valid measure of depression for Singaporean Chinese, but it does not cover the interpersonal dimension found in the AADS. Study 2 combined the two measures and found six factors. One of these factors, negative social self, which was a unique Asian depressive symptom cluster, consisted only of AADS items, while the affective manifestation and psychosomatic symptoms factor primarily consisted of items from the SDS. Study 3 selected high-loading items from the identified factors to construct the ADS, which showed excellent internal reliability, and good convergent and discriminant validity. Incremental predictive validity found on criterion data collected in stage 2, supported the nonspuriousness of the Asian Depression Scale.

## 1. Introduction

Depression is a common human condition. Subjective experiences and symptom presentation; however, vary from culture to culture [1–5]. Depressive symptoms are often expressed in “emic”—culturally conditioned “idioms of distress” [6–8]. This has led to problems in assessing depression in different cultural groups (e.g., [9–13]). The culture-depression relationship has been theorized to be mediated by the self-construal held by the individual (for instance, Markus & Kitayama [14]). The collectivist cultures of most Asian communities were found to contain relatively more interdependent self-construal (Triandis [15]); therefore, the “idiom of distress” in these communities might contain more symptoms concerning the social aspects of the self [2, 16]. 

Epidemiological studies of depression in Asians are rare and have used a variety of instruments, most of which were developed in non-Asian populations [17, 18]. These studies have reported low rates of depression in the Asia Pacific region [17]. Recently, however, observers have noted a rapid increase in reported cases of depression in Asian populations [18, 19], especially in regions such as Singapore, where there has been rapid industrial and economic development. The lack of consensus over diagnostic criteria, the lack of culturally appropriate norms [20], and the controversy over the cultural validity of the assessment tools used [6, 18] make it difficult to accurately determine the prevalence of depression in Asian regions. Culturally appropriate assessment tools for identifying depression in non-Western regions are required [17, 21]. This study constructs a comprehensive and valid measure for assessing depression in Singapore, a predominantly Chinese (75% of general population), ethnic Asian community.

## 2. Expressions of Depressive Symptoms in Asia

Earlier studies of depressive symptoms in Asian populations, especially the Chinese, have found that the Chinese tend to de-emphasize affective symptoms; instead, they present somatized complaints (e.g., [3, 5, 22, 23]). Using Zung's [24] Self-rating Depression Scale (SDS), Chang [16] found that Asian Americans showed a high salience of psychosomatic symptoms compared to other ethnic American groups. East Asian students and American students of Asian descent [10, 20, 25] exhibited depressive symptoms similar to other students, but with lower affective manifestations.

We have explored the depressive symptomatology of Asians living in Asian communities. We used in-depth interviews, followed by focus group discussions with children and adolescents. The reported depressive symptoms were factor analyzed to identify the symptom groups and to construct an Asian Adolescents Depression Scale (AADS) [26] and Asian Children's Depression Scale [27]. A common finding across both age groups was a socially-oriented dimension that contained negative feelings about self-other relationships. We think this newly identified symptom of depression—negative social Self—reflects the culture's emphasis on the interdependent self-construal in regulating one's emotions [28, 29], which is an emic expression of emotions in collectivist cultures [30]. 

The above review suggested the following features in the depressive symptoms of Asian populations: a relative de-emphasis of affective manifestations, and a strong emphasis on the somatic and interpersonal symptoms. The AADS is high on interpersonal symptoms, while SDS has a considerable portion of somatized symptoms, with considerable overlap between them. Three empirical studies were conducted to develop a comprehensive measure of depression by amalgamating these two measures.

The current study aims to establish a valid and comprehensive instrument of depression to be used mainly for screening and survey of community level screening. We have chosen the AADS and the SDS for which extensive empirical data have been collected to support each instrument's internal reliability and easy interpretability for use in Asian community samples across a wide age range. We have also considered World Health Organization's International Statistical Classification of Diseases and Related Health Problems (ICD-10) [31]. The ten-item ICD-10 taps 10 general symptoms that are considered common expressions across different cultures that are covered in Zung's SDS but does not contain the variety and diversity of each symptom category as the SDS. Beck's Depression Index (BDI) is a theory-based instrument of 21-item scale used popularly mainly in North America. The BDI has met with mixed reviews in terms of the need for multiple periods of assessments [32] and is seldom used in Asian communities [33]. We therefore chose the AADS and SDS rather than the ICD-10 and BDI.

## 3. Study 1: Internal Structure and Construct Validity of Self-Rating Depression Scale

### 3.1. Participants

Two samples were used to investigate the internal structure and the ecological validity of the SDS. The exploration sample consisted of 160 participants (39 males and 121 females, mean age 20.82, SD = 1.78 years). The validation sample consisted of 166 participants (46 males and 120 females, mean age 20.40, SD = 1.31 years). Participants were recruited from a major university in Singapore and were given course credit for their participation.

### 3.2. Instruments

#### 3.2.1. Zung's Self-Rating Depression Scale [24]

 This 20-item self-rating depression scale was developed by Zung [24] and has been widely used for both clinical and community samples. It has been used in Asian communities and was found to show acceptable reliability. A for the current study has been found to be around .80.

#### 3.2.2. The Asian Adolescent Depression Scale

 For convergent and discriminant validity: measures of positive affects (PA) [34] and measures of negative affect (NA) [34] and depression measured by the abbreviated version of Asian Adolescent Depression Scale (AADS) [26]. The AADS consists of 24 items and has shown high internal reliability. For the current sample the internal reliability was found to be *α* = .945.

#### 3.2.3. The Positive and Negative Affect Scale

The Positive and Negative Affect Scale was developed by Watson, Watson et al. [34]. This 20-item scale is divided into two subscales positive emotions (PA) and negative emotions (NA). Each consists of 10 items denoting positive emotions and negative emotions, respectively. For the present sample the internal reliability was found to be .835 and .834 for PA and NA, respectively.

#### 3.2.4. The Asian Subjective Wellbeing Scale

The Asian Subjective Wellbeing Scale was developed by Chang and Chu [35] as a measure of life satisfaction based on reported life conditions that are considered satisfying in the Singaporean Chinese community. This 30-item scale showed an internal reliability of .945.

#### 3.2.5. Test Anxiety Scale

The 20-item test anxiety scale has been widely used to assess anxiety related to being evaluated. For the present sample, the internal reliability was found to be .912.

#### 3.2.6. Hope Scale [36]

The Hope Scale constructed by Snyder et al. [36] is a 12-item measure and is divided into the hope agency (6 items) and hope pathway (6 items) subscales. For the present sample the internal reliability has been found to be .70 for the hope subscale and .65 for the pathway subscale, relatively low but acceptable.

The Life Orientation (LOT): The Life Orientation Scale [37]. The life orientation test (LOT) was developed to assess individual differences in generalized optimism versus pessimism. Using factor analysis, we trimmed off the items with insignificant loading. The remaining 9-item LOT showed a high internal reliability of .87 for the present sample. 

### 3.3. Procedures

Data were collected in two stages. In the first stage, data were collected on the SDS, the AADS, and demographic variables of the exploration sample. In the second stage, data were collected from the validation sample on a set of validation variables, including positive and negative affects such as optimism, hope, test anxiety, life satisfaction, and Asian subjective wellbeing. 

### 3.4. Results

#### 3.4.1. Exploratory Factor Analysis

 Data analysis was guided by our objective to identify underlying dimensions that are meaningful and parsimonious. To achieve this objective, we employed a series of exploratory factor analysis and conceptual grouping of items into meaningful categories to construct a hypothesized factor structural model to be subject to confirmatory factor analysis.

 First, a principal components analysis with Promax rotation was conducted on the items in the Self-rating Depression Scale. We chose Promax rotation for two reasons: (1) we hypothesized that the factors were correlated, however, (2) we did not have a apriori hypothesis as to the magnitude of factor correlation, therefore we did not use Oblimin rotation but chose Promax rotation as suggested by Gorsuch [38]. The scree plot suggested a three-factor solution, with eigenvalues and variance accounted for values being 5.80 and 29.00% for factor one; 1.84 and 9.21% for factor two; 1.40 and 7.01% for factor three, respectively. 

Factor two was labeled “loss of self-efficacy” and included items such as “I find it easy to make decisions (reversed coded)” and “I find it easy to do the things I used to do (reversed coded).” Factor three was labeled “physical symptoms” and included items such as “I eat as much as I used to (reverse coded)” and “I have trouble with constipation.” 

Additional factor analysis was conducted on Factor one, which initially included conceptually mixed items. Two conceptually coherent factors emerged: loss of life directions and affective manifestations. 

Finally, by reviewing factor loadings and sorting items into meaningful categories, four factors were identified for the SDS: loss of life direction (existentialist factor) (four items), loss of self-efficacy (five items), affective manifestations (two items), and physical symptoms (nine items).

Table 1 presents the EFA results, where LLD refers to loss of life directions, LSE refers to loss of self-esteem, AM refers to affective manifestations and PS refers to physical symptoms.

#### 3.4.2. Confirmatory Factor Analyses

The four-factor structure was tested. Each of the factors, except affective manifestations (which only has two items), was parceled. The four-factor structure had a reasonably good fit, specifically *χ* ^2^(21) = 39.24, *P* < .05, GFI = .95, AGFI = .89, and SRMR = .05.

#### 3.4.3. Internal Reliability

Internal reliability of the SDS was .801 and .803 for the exploratory sample and the validation sample, respectively. Both were acceptable, but were considerably lower than the internal reliability of .945 of AADS in the validation sample.

#### 3.4.4. Concurrent Validity

As a depression measure, the SDS should correlate substantially with other depression measures. Correlation with the AADS was *r* = .766, and *P* < .000. 

#### 3.4.5. Convergent and Discriminant Validity

As a depression measure, the SDS should correlate positively with negative emotions, anxiety, and negative affects and correlate negatively with positive emotions and positive cognitions. SDS correlated positively with negative emotions (test anxiety *r* = .547, *P* < .000 and negative affect *r* = .743, *P* < .000), and correlated negatively with positive emotions (positive affects *r* = −.445, *P* < .000 and positive cognitions, optimism (LOT), *r* = −.421, *P* < .000, hopepathway, *r* = −.348, *P* < .000, and hopegoal, *r* = −.405, *P* < .000).

#### 3.4.6. Predictive Validity

As a depression measure the SDS should predict such positive outcomes as subjective wellbeing. SDS negatively predicted subjective wellbeing measured in stage 2 (Asian Subjective Wellbeing,  *β* = −643, *t* = 10.743,   *P* < .000, and life satisfaction, *β* = .424,   *t* = 6.00,   *P* < .000).

#### 3.4.7. Discussion

The SDS correlates strongly with the AADS suggesting that it successfully assesses depression in these Asian adult samples. The EFA and CFA produced four coherent factors: Loss of life direction, loss of self-efficacy, affective manifestations, and physical symptoms. However, there were no factors that included negative social self or negative social relationships. Therefore, the SDS alone is not sufficient to assess depressive symptoms in Asian populations. 

## 4. Study 2: A Hybrid Symptomatology—Combined Items of SDS and AADS

### 4.1. Participants and Method

Participants in both subsamples were administered the SDS and AADS in random order. The items in the Self-rating Depression Scale and the Asian Adolescent Scale were combined for analyses, resulting in a total of 40 items. A series of EFA and CFA were conducted to test for the best fit for the underlying factors and the internal structure.

### 4.2. Results

#### 4.2.1. Exploratory Factor Analyses

 A principal components analysis with Promax rotation was conducted on the 40 items. The scree plot suggested a three factor solution, with eigenvalues and variance accounted for values of 13.15 and 32.88% for factor one (16 items), 2.29 and 5.72% for factor two (15 items), and 2.15 and 5.37% for factor three (nine items), respectively. 

Factor three was conceptually coherent, with items such as “I get tried for no reason,” “my heart beats faster than normal,” and “I have trouble sleeping at night.” This factor was labeled “physical symptoms.” factor one and factor two contained conceptually mixed items, so further factor analyses were conducted. 

After analysis of the 15 items in Factor One, two facets were identified: “Loss of Life Direction (existentialist factor),” which included items such as “my life is pretty full (reversed coded)” and “I feel hopeful about the future (reversed coded),” and “negative self and relationship,” which included items such as “I do not like going out with friends or meeting people” and “I feel that others would be better off if I were dead.” The remaining items consisted of symptoms of affective manifestations and were combined with factor three.

Further analysis of factor two yielded additional facets. The first and third facetswith items such as “I take a long time to decide on things” and “my mind is as clear as it used to be (reversed coded)”—were grouped into one factor, that was labeled “loss of cognitive efficacy.” The second facet included items such as “I feel that have no energy to do things most of the time” and “I do not feel like doing anything,” and was labeled “loss of motivation.” 

 In the final analysis, six factors were identified: loss of life direction (existentialist factor) (five items), negative self and relationship esteem (five items), loss of cognitive efficacy (eight items), loss of motivation (seven items), affective manifestations (five items) and physical symptoms (ten items). This six-factor solution formed the hypothesized factor model to be tested by confirmatory factor analysis.  Table 2 presents the results of EFA on the combined item pool.

#### 4.2.2. Confirmatory Factor Analysis

Finally, the six-factor structure was tested. Each of the factors was parceled. The six-factor structure had a reasonably good fit; specifically, *χ* ^2^(120) = 160.98,   *P* < .05,   GFI = .90, AGFI = .86, and SRMR = .04.

As mentioned earlier, we aimed at identifying the best fit internal structure that was meaningful and parsimonious. We hypothesized that some of the factors identified may be empirically grouped into higher order factors.

### 4.3. Identifying Higher Order Factors

#### 4.3.1. Correlations between Factors

Table 3 presents the correlations, reliabilities, and descriptive statistics of the six factors for subsample one and subsample two. The pattern of correlations between the six factors suggests that the six factors could be regrouped into three higher order factors. Loss of life direction and negative social self were highly correlated with each other in the two samples (*r*_1_ = .68, *P*_1_ < .05 and *r*_2_ = .74, *P*_2_ < .05, resp.), hence they were grouped into one higher order factor, labeled “loss of life direction”. Loss of cognitive efficacy and loss of motivation were also highly correlated with each other in both samples (*r*_1_ = .72, *P*_1_ < .05 and *r*_2_ = .78, *P*_2_ < .05, resp.) and were grouped into a second higher order factor, labeled “loss of self efficacy”. In subsample two, physical symptoms had a higher correlation with affective manifestations (*r*_2_ = .61, *P*_2_ < .05), than with the other factors, hence, these two factors were grouped into a third higher order factor, labeled “affective and physical symptoms.” 

Confirmatory factor analyses were then conducted to test and confirm the conceptual appropriateness of the three-factor structure of depression.

**Subsample One**

Using the item mean of each factor as a manifested indicator, with two indicators for each of the three latent factors, a reasonably good fit was found for the higher order structure (*χ* ^2^(6) = 22.70, *P* < .05, GFI = .96, AGFI = .85, SRMR = .04).

**Subsample Two**

Using the item mean of each factor as an indicator, with two indicators for each of the three latent factors, a good fit was found for the higher order structure (*χ* ^2^(6) = 6.83, *P* = ns, GFI = .99, AGFI = .95, SRMR = .02).

### 4.4. Discussion

Of the six factors that emerged from the joint item pool, the negative social self factor, which consists mainly of AADS items, appears to be a specifically Asian depressive manifestation. The loss of self-efficacy factor, which includes loss of cognitive efficacy and loss of motivation symptoms, corresponds with young Asian's worries about achievement and failure. The SDS's factor of affective and somatic symptoms confirms the earlier findings on Asian depressive complaints. The integrated symptomatology of AADS and SDS is a more comprehensive reflection of the contemporary Asian experience of depression [18] than either AADS or SDS alone. 

## 5. Study 3: An Asian Depression Measure

Using the integrated symptomatology of depression derived in Study 2, we constructed the Asian Depression Scale (ADS) and assessed its internal reliability, construct validity, and incremental validity. 

### 5.1. Participants and Method

Data from the validation sample were used to construct and validate the ADS. Materials used were as described above.

#### 5.1.1. Scale Construction

We examined the individual item loadings in all three factors and chose the higher loading items and deleted redundant items. The resultant scale maintained the comprehensive factor structure of the integrated symptomatology.

#### 5.1.2. Data Analysis

We explored the internal reliability, the convergent and predictive validity of the newly constructed ADS. We conducted exploratory factor analysis followed by confirmatory factor analysis (EFA and CFA) for the ADS. Finally, using validating data collected at stage 2, we tested whether the incremental validity of the ADS was a spurious measure over SDS or AADS.

### 5.2. Results

#### 5.2.1. Confirmatory Factor Analysis

Table 4 presents the items and factors of the Asian Depression Scale. Using the 24-item ADS, the three-factor structure yielded a reasonable fit (*χ* ^2^(249) = 403.84, *P* < .05, GFI = .83, AGFI = .79, SRMR = .06), lending empirical support to the validity of loss of meaning of life, the loss of self-efficacy, and affective-somatic symptoms as dimensions constituting the ADS (see Table 3). 

#### 5.2.2. Internal Reliability

 The ADS had excellent psychometric properties. Cronbach's alpha was .964. 

#### 5.2.3. Concurrent Validity

The intercorrelation between the ADS with the AADS were *r* = .980  and  *P* < .000, and SDS, *r* = .861 and *P* < .000; both significantly high, suggesting that the ADS measures the same underlying construct as AADS and SDS.

#### 5.2.4. Discriminant and Convergent Validity

 The ADS was negatively correlated with positive affects and cognitions. It was negatively correlated with positive affect (*r* = −.502, *P* < .000); hope pathway (*r* = −.391, *P* < .000); hope agency (*r* = −.483, *P* < .000); optimism (*r* = −.403, *P* < .000). Furthermore, the ADS was positively correlated with negative affect (*r* = .865, *P* < .000) and test anxiety (*r* = .541, *P* < .000). 

#### 5.2.5. Predictive Validity

The SDS predicted subjective wellbeing, as measured in stage 2. The SDS negatively predicted both life satisfaction (*β* = − .540, *R* ^2^ = .292, *P* < .00) and Asian subjective wellbeing (*β* = −.726, *R* ^2^ = .527, *P* < .00), contributing a higher percentage of variance (23%) to Asian subjective wellbeing than to life satisfaction. 

#### 5.2.6. Incremental Validity

 We assessed the incremental validity of the ADS for each criterion measure, including Asian subjective wellbeing, life satisfaction, positive affect, negative affect, and test anxiety compared to the SDS and AADS. When the AADS was parceled out, the ADS made a marginally significant contribution to Asian subjective wellbeing (Δ*R* ^2^ = .009, *F* = 3.517, *df* = 163, *P* = .063); to life satisfaction (Δ*R* ^2^ = .003, *F* = .776, *df* = 163, *n*.*s*.); to positive affect (Δ*R* ^2^ = .009, *F* = 3.517, *df* = 163, *P* = .063), suggesting a small incremental validity of the ADS over AADS on positive criteria. The ADS contributed independently to test anxiety with marginal significance (Δ*R* ^2^ = .015, *F* = 3.430, *df* = 163, *P* = .066) and to negative affect (Δ*R* ^2^ = .002, *F* = 1.091, *df* = 163, *P* = .298), suggesting a small incremental validity of ADS over AADS on negative criteria.

When the SDS was controlled for, ADS independently contributed to Asian subjective wellbeing, (Δ*R* ^2^ = .015, *F* = 55.904, *df* = 163, *P* < .000); to life satisfaction (Δ*R* ^2^ = .015, *F* = 36.617, *df* = 163, *P* < .000); to positive affect (Δ*R* ^2^ = .023, *F* = 4.752, *df* = 163, *P* = .031), suggesting the significant incremental validity of the ADS over SDS on positive criteria; furthermore, the ADS contributed to negative affect (Δ*R* ^2^ = .017, *F* = 96.762, *df* = 163, *P* < .000) and to test anxiety (Δ*R* ^2^ = .028, *F* = 6.799, *df* = 163, *P* = .010), suggesting a significant incremental validity of the ADS over the SDS on negative criteria. 

### 5.3. Discussion

The ADS demonstrates excellent internal reliability and is highly correlated with the ADS and the SDS. Furthermore, the ADS correlates with both positive and negative affects, subjective wellbeing and hope, and life satisfaction in the theoretically correct directions. This pattern of correlations supports the conclusion that the ADS is a measure of depression. Most importantly, the ADS demonstrates significant incremental validity over the AADS and SDS in predicting positive and negative outcomes, suggesting that it is not a spurious measure of depression for the Asian population. 

## 6. General Discussion

The amalgamated items from the SDS and the AADS have excellent internal coherence and culturally meaningful factors, suggesting that the combined list provides a more accurate assessment of depression for Singaporean adults than either measure alone. 

The ADS yields incremental validity on the positive and negative criteria over its source measures, suggesting that it is not a spurious measure of depression over the AADS or the SDS. 

These empirical studies were conducted with Singaporean Chinese samples; however, this should not greatly limit the application of the ADS. The social dimension identified here reflects a shared Asian cultural emphasis on the interdependence of self-construal. The ADS is more suitable for other collectivist Asian cultures than the standardized Western measures. 

Cross-cultural comparisons with non-Asian populations should also be conducted to test the boundary of cultural limitations of the instrument. Furthermore, as the ADS was developed on community samples, studies should be conducted to test the utility of the scale in clinical settings. 

## Figures and Tables

**Table 1:** Factor structure and factor loading of self-rating depression scale.

Item number	Item	LLD	LSE	AM	PS
S 14	I feel hopeful about the future	0.57			
S 17	I feel that I am useful and needed	0.8			
S 18	My life is pretty full	0.71			
S 19	I feel that others would be better off if I were dead	0.54			
S 11	My mind is as clear as it used to be		0.69		
S 12	I find it easy to do the things I used to do		0.7		
S 16	I find it easy to make decisions		0.43		
S 2	Morning is when I feel the best		0.17		
S 20	I still enjoy the things I used to do		0.45		
S 1	I feel sad			0.71	
S 3	I have crying spells or feel like it			0.87	
S 4	I have trouble sleeping at night				0.47
S 5	I eat as much as I used to				0.26
S 6	I enjoy sex				0.24
S 7	I notice that I am losing weight				0.22
S 8	I have trouble with constipation				0.45
S 9	My heart beats faster than normal				0.65
S 10	I get tried for no reason				0.55
S 13	I am restless and cannot keep still				0.08
S 15	I am more irritable than usual				0.42

Note. “S” refers to items in the Self-rating Depression Scale; LLD is loss of life direction; LSE is loss of self-efficacy; AM is affective manifestations; PS is physical symptoms.

**Table 2:** Factor structure and factor loading of Self-Rating Depression Scale and Asian Adolescent Depression Scale.

Item number	Item	LLD	NSS	LCE	LOM	AM	PS
S 14	I feel hopeful about the future	0.6					
S 17	I feel that I am useful and needed	0.71					
S 18	My life is pretty full	0.66					
A 3	I feel that I am not wanted	0.71					
A 5	I feel hopeless	0.66					
S 19	I feel that others would be better off if I were dead		0.61				
A13	I do not like going out with friends or meeting people		0.53				
A 16	I have thought about dying		0.54				
A 19	I feel that I am not as good as others		0.77				
A 20	Nothing works out right for me		0.75				
S 11	My mind is as clear as it used to be			0.49			
S 12	I find it easy to do the things I used to do			0.43			
S 16	I find it easy to make decisions			0.66			
A 4	I take a long time to get things done			0.76			
A 8	I cannot think well			0.73			
A 11	I cannot concentrate on my studies as much as I used to			0.6			
A 12	I take a long time to decide on things			0.76			
A 18	I am confused about what kind of person I am			0.67			
S 2	Morning is when I feel the best				0.3		
S 20	I still enjoy the things I used to do				0.16		
A 6	I feel tired most of the time				0.73		
A 9	I do not feel like doing anything				0.77		
A 14	I feel that I have no energy to do things most of the time				0.7		
A 15	I do not get satisfaction from what I do				0.72		
A 17	I feel that I have no control over what happens				0.63		
S 1	I feel sad					0.61	
S 3	I have crying spells or feel like it					0.6	
A 1	I feel sad most of the time					0.76	
A 2	My heart feels heavy					0.74	
A 10	I often feel like crying					0.6	
S 4	I have trouble sleeping at night						0.45
S 5	I eat as much as I used to						0.18
S 6	I enjoy sex						0.29
S 7	I notice that I am losing weight						0.12
S 8	I have trouble with constipation						0.35
S 9	My heart beats faster than normal						0.53
S 10	I get tried for no reason						0.65
S 13	I am restless and can't keep still						0.11
S 15	I am more irritable than usual						0.53
A 7	I am more bad tempered than before						0.55

Note. “S” refers to items in the Self-rating Depression Scale; “A” refers to items in the Asian Adolescent Depression Scale. LLD: loss of life direction; NSS: negative social self; LCE: loss of cognitive efficacy; LOM: loss of motivation; AM: affective manifestations; PS: physical symptoms.

**Table 3:** Correlations, reliabilities, and descriptive statistics of the six factors for the Subsample one (English) and Subsample two.

Factors	LLD	NSS	LCF	LOM	AM	PS
Subsample one						

LLD	1.00					
NSS	.68**	1.00				
LCF	.67**	.56**	1.00			
LOM	.66**	.61**	.72**	1.00		
AM	.65**	.62**	.52**	.51**	1.00	
PS	.60**	.44**	.59**	.64**	.52**	1.00
*α*	.87	.76	.84	.78	.88	.66
M	2.39	2.14	2.78	2.70	2.43	2.54
SD	.77	.70	.68	.63	.80	.52

Subsample two						

LLD	1.00					
NSS	.74**	1.00				
LCF	.74**	.68**	1.00			
LOM	.73**	.42**	.78**	1.00		
AM	.65**	.69**	.64**	.65**	1.00	
PS	.55**	.56**	.49**	.56**	.61**	1.00
*α*	.81	.77	.85	.78	.84	.62
M	2.24	2.14	2.81	2.54	2.39	2.54
SD	.59	.70	.71	.64	.74	.45

Note. LLD: loss of life direction; NSS: negative social self; LCF: loss of cognitive efficacy; LOM: loss of motivation; AM: affective manifestations; PS: physical symptoms.

**P < .01.

**Table 4:** The Asian Depression Scale.

Factor one: loss of meaning of life	
18	I feel that I am not wanted
5	I feel hopeless
15	I feel that others would be better off if I were dead
4	I do not like going out with friends or meeting people
17	Nothing works out right for me

Factor two: loss of self-efficacy	

22	My mind is as clear as it used to be
23	I find it easy to do the things I used to do
19	I find it easy to make decisions
7	I cannot think well
9	I cannot concentrate on my studies as much as I used to
20	I take a long time to decide on things
3	I am confused about what kind of person I am
2	I feel tired most of the time
13	I do not feel like doing anything
8	I feel that I have no energy to do things most of the time
6	I do not get satisfaction from what I do
16	I feel that I have no control over what happens

Factor three: Affective-somatic symptoms	

21	I feel sad
11	I have crying spells or feel like it
14	My heart feels heavy
1	I often feel like crying
24	My heart beats faster than normal
10	I get tried for no reason
12	I am more bad tempered than before

Note: Numbers refer to item numbering in the Asian Depression Scale.

## References

[1] Chentsova-Dutton YE, Tsai JL, Ingram RE (2009). Understanding depression across cultures. *International Encyclopedia of Depression*.

[2] Essau CA, Chang WC, Essau CA (2009). Epidemiology, co-morbidity and course of adolescent depression. *Treatments for Adolescent Depression*.

[3] Kleinman A (1980). *Patients and Healers in the Context of Culture: An Exploration of the Borderland between Anthropology, Psychiatry and Medicine*.

[4] Kleinman A (2004). Culture and depression. *The New England Journal of Medicine*.

[5] Kleinman A, Good BJ, Kleinman A, Good BJ (1985). Introduction: culture and depression. *Culture and Depression: Studies in the Anthropology and Cross-Cultural Psychiatry of Affect and Disorder*.

[6] Marsella AJ (1978). Thoughts on cross-cultural studies on the epidemiology of depression. *Culture, Medicine and Psychiatry*.

[7] Patel V (2001). Cultural factors and international epidemiology. *British Medical Bulletin*.

[8] Tsai JL, Chentsova-Dutton Y, Gotlib IH, Hammen CL (2002). Understanding depression across cultures. *Handbook of Depression*.

[9] Barbee EL (1992). African american women and depression: a review and critique of the literature. *Archives of Psychiatric Nursing*.

[10] Iwata N, Buka S (2002). Race/ethnicity and depressive symptoms: a cross-cultural/ethnic comparison among university students in East Asia, North and South America. *Social Science and Medicine*.

[11] Malik R, Squire C (2000). Culture and emotions: depression among Pakistanis. *Culture in Psychology*.

[12] Obeyesekere G, Kleinman A, Goode BJ (1985). Depression, Buddhism and the Work of Culture in Sri Lanka. *Culture and Depression*.

[13] Phan T, Steel Z, Silove D (2004). An ethnographically derived measure of anxiety, depression and somatization: The Phan Vietnamese psychiatric scale. *Transcultural Psychiatry*.

[14] Markus HR, Kitayama S (1991). Culture and the self: implications for cognition, emotion and motivation. *Psychological Review*.

[15] Triandis HC (1989). The self and social behavior in differing cultural contexts. *Psychological Review*.

[16] Chang WC (1985). A cross-cultural study of depressive symptomology. *Culture, Medicine and Psychiatry*.

[17] Chiu E, Hickie I (2004). Epidemiology of depression in the Asia Pacific region. *Australasian Psychiatry*.

[18] Lim LL, Chang W, Yu X, Chiu H, Chong MY, Kua EH (2011). Depression in Chinese elderly populations. *Asia-Pacific Psychiatry*.

[19] Chen JP, Chen H, Chung H (2002). Depressive disorders in Asian American adults. *Western Journal of Medicine*.

[20] Crittenden KS, Fugita S, Bae H, Lamung C, Un C (1992). A cross-cultural study of self-report depressive symptoms among college students. *Journal of Cross-Cultural Psychology*.

[21] Leong FTL, Okazaki S, Tak J (2003). Assessment of depression and anxiety in East Asia. *Psychological Assessment*.

[22] Parker G, Gladstone G, Kuan Tsee Chee (2001). Depression in the planet’s largest ethnic group: The Chinese. *American Journal of Psychiatry*.

[23] Yeung A, Kam R (2006). Recognizing and treating depression in Asian Americans. *Psychiatric Times*.

[24] Zung WW (1965). A self-rating depression scale. *Archives of General Psychiatry*.

[25] Fugita SS, Crittenden KS (1990). Towards culture- and population-specific norms for self-reported depressive symptomatology. *International Journal of Social Psychiatry*.

[26] Woo BSC, Chang WC, Fung DSS (2004). Development and validation of a depression scale for Asian adolescents. *Journal of Adolescence*.

[27] Koh JBK, Chang WC, Fung DSS, Kee CHY (2007). Conceptualization and manifestation of depression in an Asian context: formal construction and validation of a children’S depression scale in Singapore. *Culture, Medicine and Psychiatry*.

[28] Chang WC, Lee L (2012). The concentric circle revisited: relationship and self-representations in a modern Chinese community. *Psychology*.

[29] Lam BT (2005). Self-construal and depression among Vietnamese-American adolescents. *International Journal of Intercultural Relations*.

[30] Chentsova-Dutton YE, Tsai JL (2010). Self-focused attention and emotional reactivity: the role of culture. *Journal of Personality and Social Psychology*.

[31] World Health Organization (2010). *Major (ICD-10) Depression Inventory*.

[32] Kendall PC, Hollon SD, Beck AT (1987). Issues and recommendations regarding use of the beck depression inventory. *Cognitive Therapy and Research*.

[33] Yeung A, Howarth S, Chan R, Sonawalla S, Nierenberg AA, Fava M (2002). Use of the Chinese version of the beck depression inventory for screening depression in primary care. *Journal of Nervous and Mental Disease*.

[34] Watson D, Clark LA, Tellegen A (1988). Development and validation of brief measures of positive and negative affect: The PANAS Scales. *Journal of Personality and Social Psychology*.

[35] Chang WC, Chu R Asian subjective wellbeing: conceptualization and opertionalization.

[36] Snyder CR, Sympson SC, Ybasco FC, Borders TF, Babyak MA, Higgins RL (1996). Development and validation of the state hope scale. *Journal of Personality and Social Psychology*.

[37] Carver CS, Scheier MF, Segerstrom SC (2010). Optimism. *Clinical Psychology Review*.

[38] Gorsuch RL (1997). Exploratory factor analysis: Its role in item analysis. *Journal of Personality Assessment*.

